# Tanshinones induce tumor cell apoptosis via directly targeting FHIT

**DOI:** 10.1038/s41598-021-91708-z

**Published:** 2021-06-09

**Authors:** Xianglian Zhou, Yuting Pan, Yue Wang, Bojun Wang, Yu Yan, Yi Qu, Xisong Ke

**Affiliations:** 1 Center for Chemical Biology, Institute of Interdisciplinary Integrative Medicine Research, Shanghai University of Traditional Chinese Medicine, Shanghai, China; 2 Shanghai Innovation Center of TCM Health Service, Shanghai University of Traditional Chinese Medicine, Shanghai, China

**Keywords:** Biochemistry, Cell biology

## Abstract

The liposoluble tanshinones are bioactive components in *Salvia miltiorrhiza* and are widely investigated as anti-cancer agents, while the molecular mechanism is to be clarified. In the present study, we identified that the human fragile histidine triad (FHIT) protein is a direct binding protein of sodium tanshinone IIA sulfonate (STS), a water-soluble derivative of Tanshinone IIA (TSA), with a Kd value of 268.4 ± 42.59 nM. We also found that STS inhibited the diadenosine triphosphate (Ap3A) hydrolase activity of FHIT through competing for the substrate-binding site with an IC_50_ value of 2.2 ± 0.05 µM. Notably, near 100 times lower binding affinities were determined between STS and other HIT proteins, including GALT, DCPS, and phosphodiesterase ENPP1, while no direct binding was detected with HINT1. Moreover, TSA, Tanshinone I (TanI), and Cryptotanshinone (CST) exhibited similar inhibitory activity as STS. Finally, we demonstrated that depletion of FHIT significantly blocked TSA’s pro-apoptotic function in colorectal cancer HCT116 cells. Taken together, our study sheds new light on the molecular basis of the anti-cancer effects of the tanshinone compounds.

## Introduction

The human fragile histidine triad (FHIT) is a tumor suppressor gene located at chromosome region 3p14.2, which inactivated in > 50% human tumors accounting for deletion, mutation, and hypermethylation^1^. FHIT is a member of the histidine triad (HIT) family with a conserved histidine triad sequence motif at the N-terminal that are important for the diadenosine triphosphate (Ap3A) binding and hydrolysis^2–4^. Previous studies revealed that re-expression of FHIT in FHIT-negative tumor cells resulted in significant cell proliferation inhibition^5,6^. Recent studies showed that the function of FHIT in tumor suppression depends on Ap3A binding^7,8^. Because FHIT H96N mutant without hydrolase activity still has anti-tumor functions, while L25W mutant without Ap3A binding activity shows no tumor suppressive function. In line with a tumor suppressor function, FHIT loss increased the migration and invasion capacities of tumor cells^9–11^. Various signaling pathways were altered upon FHIT loss or overexpression, such as NF-κB signaling^12^, Ras/Rho GTPase signaling^13^, DNA damage-response checkpoint activation^14^, ROS production^15,16^.

A few molecules have been reported to date that are able to inhibit FHIT’s enzymatic activity. Among these, suramin and non-cleavable ApnA analogues have been presented over a decade which inhibit FHIT without selectivity compared to other different classes of enzymes^17,18^. During past year, a high-throughput screening assay of Förster Resonance Energy Transfer (FRET) was developed to discover potent small molecule inhibitors of FHIT based on a doubly labeled Ap3A probe^19^, and several inhibitors with methylpyrazolo-motive or indino-motive were identified to block FHIT activity^20^, but those inhibitors were not applied to cellular content, potentially due to the difference of protein between purified protein and cellular protein with extensive post-translational modification and protein–protein interactions^21^.

*Salvia miltiorrhiza (SM)* is a widely used traditional Chinese medicine in China^22^. The main active ingredients of *SM* are liposoluble tanshinone compounds, which mainly include tanshinone IIA (TSA), dihydrotanshinone (DT), tanshinone I (TanI), and cryptotanshinone (CT)^23,24^. Among these compounds, TSA has been broadly investigated due to its various biological activities, especially cancer inhibition^25–28^. Recently, TSA’s anti-cancer activities were connected with induction of apoptosis and autophagy, inhibition of cell growth and migration by regulating AMPK^29,30^, Notch1/NF-κB^31^, PI3K/Akt/mTOR signaling pathway^32^, and so on. Sodium tanshinone IIA sulfonate (STS), a water-soluble sulfonate on C-16 position derived from TSA, has been approved to treat cardiovascular diseases for more than a decade^33^. Numerous studies demonstrated that the beneficial effects of STS in various diseases are attributable to its role in reducing reactive oxygen species (ROS) production and decreasing pro-inflammatory cytokines^34–38^.

Here, we report that STS directly engaged to FHIT protein and inhibited FHIT Ap3A hydrolase activity through the competitive binding to the FHIT substrate binding site. Similar effects were observed with other tanshinone analogs, including TSA, TanI, and CST, although STS and TSA were very different on cell toxicity. Finally, we demonstrated that FHIT depletion partially blocked the pro-apoptotic function of TSA.

## Results

### STS directly binds to FHIT protein

**Figure 1:**
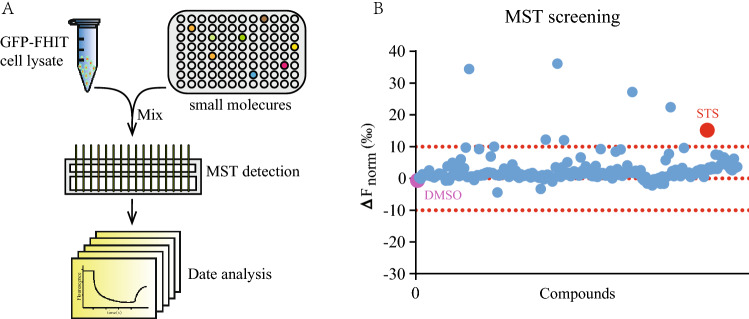
MST screening of small molecules binding FHIT. (**A**) Schematic overview of MST based single-point screening. (**B**) Plotting of single-point MST screening of a library of FDA products towards FHIT.

**Figure 2:**
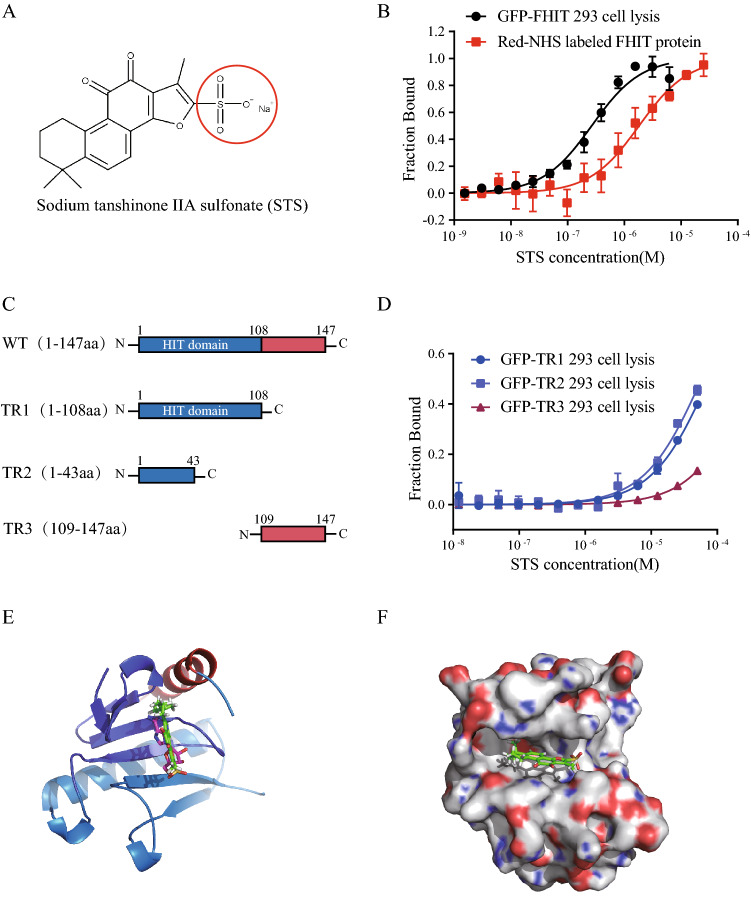
STS directly binds to FHIT protein. (**A**) Chemical structure of STS. (**B**) Cellular MST assay of the interaction between STS and FHIT. HEK293T cell lysate containing GFP tagged FHIT (black circle) or Red-NHS labeled FHIT (red square) were incubated with STS. The Kd value is 268.4 ± 42.59 nM and 1.82 ± 0.3 µM for GFP-FHIT and Red-NHS FHIT, respectively. (**C**) Schematic representation of the full-length FHIT and the truncated mutants used in the MST assay. The N-terminal region (1-108aa) containing the HIT domain is colored by blue, and the C terminal is colored by red. (**D**) MST assay of STS and GFP tagged FHIT truncations, the Kd value is 75.05 µM for TR1 (blue circle), and 58.18 µM for TR2 (blue square). No Kd value was calculated with TR3 (dull-red triangle). (**E**) Representation of molecule docking of FHIT-STS complex by Autodock vina software based on the FHIT-AMP complex (PDB code:3FIT). The region of 1-43aa is shown in mazarine, 44-108aa is shown in wathet, 109-147aa is shown in red. STS is shown in green sticks and AMP is shown in purple sticks. Structure alignment indicated that STS almost occupies the AMP binding site. (**F**) The surface model of the predicated FHIT-STS complex.

To identify small molecule directly binding to FHIT in cellular content, we took the advantage of microscale thermophoresis (MST) assay that allows determining the direct engagement between small molecule and cellular target proteins in biological liquid^39,40^. We first created HEK293T cells containing GFP-tagged FHIT and prepared the cell lysates for single-point MST binding check screening. In a FDA-approved drug library containing 1971 compounds, several small molecules were found directly binding to FHIT (Fig. 1). Among these candidates, MST binding affinity check assay revealed small molecule with the highest binding affinity is STS, a water-soluble derivative of TSA, and the Kd value between STS and FHIT was 268.4 ± 42.59 nM (Fig. 2B). To further verify the direct interaction between STS and FHIT, we purified FHIT protein in *E.coli.* Interestingly, we also detected a binding curve using the purified FHIT, but the binding affinity (Kd value = 1.82 ± 0.3 µM) was 5 times lower that the cellular FHIT (Fig. 2B). The higher binding affinity of cellular FHIT and STS supports the advantage of drug screening using cellular protein.

### STS binds to the FHIT substrate-binding site

The overall structure of FHIT is displayed as a general α + β type, and the N terminal is the HIT domain which accounts for Ap3A binding and hydrolysis (Fig. 2C). The FHIT protomer contains two helices, A and B, and seven β strands^41^. Substrate binds on the surface of the pocket consisted of seven β strands in the crystal structure of the FHIT-IB2 complex^7^. To determine the binding site of STS, we constructed three GFP-tagged FHIT truncations (Fig. 2C). In cellular MST assay, robust binding curve was determined with STS and truncation region 1 (TR1) and TR2, but no direct binding was observed with TR3, which represented C-terminal only (Fig. 2D). The occupation of STS to the HIT domain implies that STS potentially attaches to the substrate-binding pocket of FHIT. To validate the possibility, molecule docking was proposed to assess the interaction between STS and FHIT using the published crystal structure of the FHIT-AMP complex [Protein Data Bank (PDB): 3FIT]. Indeed, the prediction showed that STS adopts a similar three-dimensional (3D) conformation upon docking to FHIT as AMP (Fig. 2E). Moreover, STS could be well fitted into the ‘active site’ of FHIT in the surface model (Fig. 2F).

### STS competitively inhibits FHIT hydrolase activity

**Figure 3:**
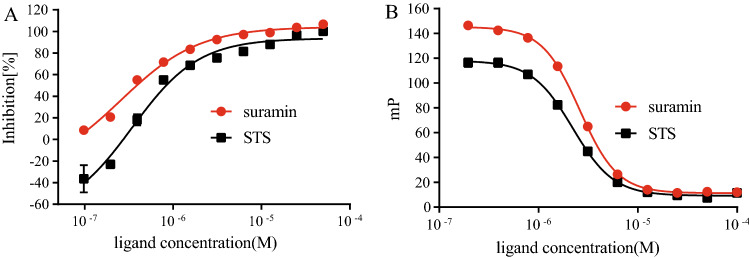
STS inhibits FHIT hydrolase activity through occupation of FHIT substrates binding pocket. (**A**) STS significantly inhibited FHIT Ap3A hydrolase activity (black square) with IC50 = 0.34 ± 0.09 µM, the IC50 of suramin (red circle) was 0.26 ± 0.08 µM. (**B**) In fluorescence polarization assay, the IC50 was 2.2 ± 0.05 µM for STS (black square) and FHIT H96N-ApppBODIPY binding inhibition, and the IC50 was 2.6 ± 0.044 µM for suramin (red circle).

Next, we attempted to assess whether STS inhibits the hydrolase activity of FHIT. It has been established that Ap3A can be rapidly hydrolyzed to AMP and ADP by FHIT^2^. Therefore the production of ADP in the reaction system could represent the hydrolase activity of FHIT. With this principle, we determined the IC50 value of suramin (a known inhibitor of FHIT) is 0.26 ± 0.08 µM, which was comparable to previous report^17^. Using the same method, we found that STS inhibits the hydrolase activity of FHIT with an IC50 value of 0.34 ± 0.09 µM (Fig. 3A). To further investigate whether STS inhibits FHIT hydrolase activity via competitive engagement with the substrate-binding site of FHIT, we purified an FHIT mutant that substituted His to Asn at the central histidine (H96N mutant), which is catalytically inactive with no effect on substrate binding^7,8^. Consistently, in a fluorescence polarization assay with the H96N mutant utilizing a fluorogenic substrate ApppBODIPY, STS significantly disrupted the interaction between H96N mutant and ApppBODIPY with an IC50 value of 2.2 ± 0.05 µM (Fig. 3B). Similar result was observed for suramin with an IC50 value of 2.6 ± 0.044 µM (Fig. 3B). All those results indicated that STS is a competitive inhibitor for FHIT.

### STS binds to HIT family proteins with increased Kd values and inhibits ENPP1 hydrolase activity at higher concentration

**Figure 4:**
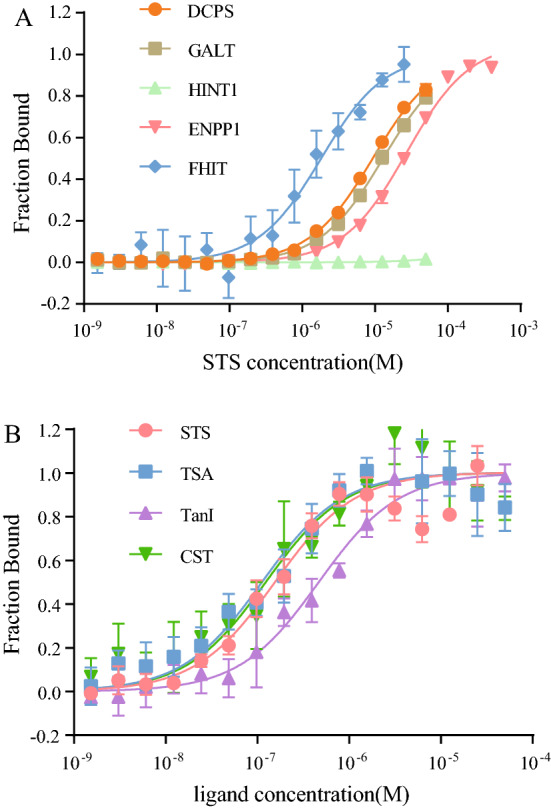
Selectivity assay of the binding between STS and FHIT. (**A**) The binding affinity determination between STS and purified HIT family proteins by MST, and the Kd value was 9.31 ± 0.4 µM for DCPS (orange circle), 12.92 ± 0.54 µM for GALT (celadon square), and 27.22 ± 1.96 µM for ENPP1 (pink inverted triangle). No binding affinity was calculated with HINT1 (light green triangle). FHIT (blue rhombus) was used as a control. (**B**) MST assay of the binding between tanshinone compounds and FHIT, the Kd value is 112 ± 27.3 nM for TSA (blue square), 466 ± 60 nM for TanI (purple triangle), and 123 ± 31.7 nM for CTS (green inverted triangle). STS (orange circle) was used as a control.

Totally there are seven HIT proteins and classified into five branches according to their structure and functions in humans^42^. To investigate the selectivity of STS to those HIT proteins, we purified other three HIT proteins, HINT1, DCPS, and GALT. It was reported that ENPP1 rapidly hydrolyzed Ap3A into ADP and AMP, thereby regulating the function of platelet^43^. As expected, direct binding was also observed between STS and ENPP1, although different binding affinities were detected for those HIT proteins and ENPP1, for examples, the Kd value for DCPS is 9.31 ± 0.4 µM, for GALT is 12.92 ± 0.54 µM, for ENPP1 is 27.22 ± 1.96 µM (Fig. 4A). Notably, no direct binding curve was observed between STS and HINT1 (Fig. 4A). We also tested whether STS inhibits Ap3A hydrolyze activity of ENPP1. In the same method of Ap3A hydrolase activity detection as described above (Fig. 3A), STS showed much less inhibition to ENPP1 compared to suramin (Fig. S1B). This was confirmed by the thin layer chromatography (TLC) assay with ApppBODIPY, in that suramin inhibited ENPP1 hydrolase activity at 6.25 µM, whereas STS did not show inhibition until to 125 µM (Fig. S1C). Altogether, These results indicate that STS specifically inhibits FHIT hydrolase activity compared with ENPP1. The inhibition of STS on the enzyme activity of DCPS and GALT needs further investigation.

### STS analogs inhibit FHIT enzyme activity

**Figure 5:**
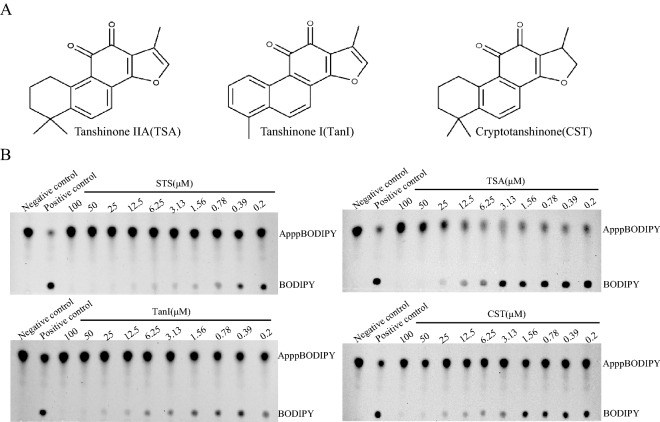
Tanshinone compounds inhibit FHIT Ap3A hydrolase activity. (**A**) Chemical structure of Tanshinone IIA (left), Tanshinone I (middle), and Cryptotanshinone (right). (**B**) Fluorescent thin layer enzyme assay, significant inhibition activity was detected in all the tanshinone compounds with IC50 value at ~ 5 µM.

There are two other tanshinone compounds TanI and CST with similar biological effects as TSA, the major bioactive compound of Danshen^44^. Moreover, the structures of these three compounds are very similar, except that CST has a single bond at the C-15 position of the furan ring and TanI having a methylbenzene instead of dimethyl cyclohexanes at the left compared with TSA (Fig. 5A). To investigate whether these three compounds inhibit FHIT hydrolase activity or not, we firstly determined the binding affinity between those compounds and FHIT via MST assay. As expected, all the compounds displayed similar binding curves with the Kd value around 100–150 nM except TanI (Fig. 4B). The Kd value of TanI increased by four-folds (466 ± 60 nM), which may be due to the different structure of TanI at the left compared with others. Furthermore, in TLC assay with ApppBODIPY^45^, TSA, TanI, and CST exhibited similar inhibition on FHIT hydrolase activity with IC50 values of 4 ~ 6 µM, and STS displayed slightly more effective inhibition than others (Fig. 5B).

### The effect of TSA inducing tumor cell apoptosis is blocked by FHIT depletion

**Figure 6:**
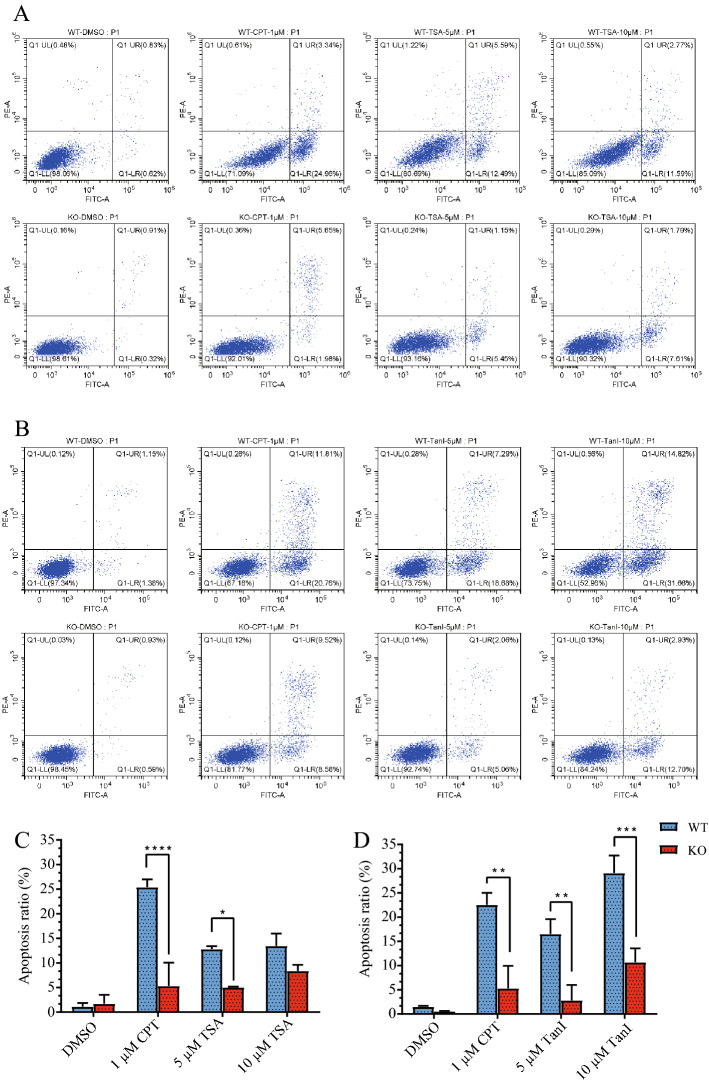
TSA inhibition cell growth is partially dependent on FHIT. Flow cytometry assay of apoptosis analysis for TSA (**A**) and TanI (**B**), FHIT WT and KO HCT116 cells were treated with the indicated concentrations of ligand for 24 h, then cells were stained for PI and Annexin V before flow cytometry analysis. (**C**) Qualification of V-FITC/PI measurements of apoptotic cells in (**A**). The experiment was repeated three times in each group. Data were analyzed using two-way ANOVA, one asterisk stands for P < 0.05, four asterisks stand for P < 0.0001. (**D**) Quantification of V-FITC/PI positive apoptotic cells in (**B**). The experiment was repeated 3 times in each group. Data were analyzed using two-way ANOVA, two asterisks stand for P < 0.001, three asterisks stand for P < 0.0005.

It has been reported that the FHIT-Ap3A complex may mediate the anti-tumor function of FHIT by inducing apoptosis^7,8^. To investigate whether tanshinone compounds inhibit tumor cell growth via FHIT-dependent apoptosis pathway, we created FHIT knockout (KO) colorectal HCT116 cells by CRISPR/Cas9 mediated genome editing, genomic DNA sequence revealed that exon5 to exon6 of the FHIT gene was deleted (Fig. S2A), and the depletion of FHIT protein was confirmed by western blotting (Fig. S2B). In ordinary culture, no clear change of FHIT knockout cells was observed regarding the epithelial morphology and growth rate compared to wild-type (WT) cells (Fig. S2D). Next, we determined the cytotoxicity of STS and TSA in WT HCT116 cells. CCK8 assay shown STS had no cytotoxicity, even the concentration is as high as 100 µM (Fig. S3A). TSA significantly inhibits the growth of HCT116 cells at 2 µM (Fig. S3A). A previous study reported that TSA, TanI, and CST induced tumor cell apoptosis^46^. To ask if FHIT knockout disrupt the pro-apoptotic effects of TSA and TanI, apoptosis was detected by flow cytometry with Annexin V-fluorescein isothiocyanate (V-FITC) and propidium iodide (PI) staining. When cells were treated with 5 µM TSA for 24 h, the apoptosis rate (4.9%) in FHIT KO cells was significantly lower than the rate (13.5%) in WT cells (Fig. 6A). Similar to previous report^47^, when cells were treated with camptothecin (CPT) at 1 µM that served as a positive control, apoptosis was significantly lower in KO cells (5.3%) than in WT cells (24.9%) as well (Fig. 6C). Notably, TanI led more striking difference in apoptosis assay between WT and KO cells, in that TanI at 10 µM induced 31.66% apoptosis in WT cells and 12.7% apoptosis cells in KO cells (Fig. 6B), a significant difference was also presented at 5 µM (Fig. 6D). All these findings indicated that tanshinone compounds induction of tumor cell apoptosis is partially dependent on FHIT.

## Discussion

Tanshinones are natural terpenoids and the main bioactive ingredients of *Salvia miltiorrhiza*^23,24^. Previous studies showed that tanshinones inhibit tumor via induction of various apoptosis signaling pathways^46^. Our study demonstrated that tanshinone compounds directly bind to FHIT and inhibit the hydrolase activity. We found that STS is a competitive inhibitor of FHIT by occupying the ‘active site’ with a IC_50_ value of 2.2 ± 0.05 µM. As the substrate-binding activity of FHIT is essential for its anti-tumor function^7,8^, we speculate that STS might mimic the function of Ap3A. Given that FHIT-Ap3A complex was immediately abolished when Ap3A was hydrolyzed^48^, while STS functions as a nonhydrolyzable substrate, the stabilized FHIT-STS complex could enhance the anti-tumor function of FHIT. In addition, it has been reported that a nonhydrolyzable analog of Ap4A binded to FHIT and stabilized its’ flexible loop which was important for its tumor suppression activity^49^. Therefore, STS may function in a similar way to enhances the anti-tumor function of FHIT as the nonhydrolyzable substrate. Moreover, pro-apoptotic induction by TSA and TanI’s was partially blocked by FHIT depletion. Considering that FHIT knockout did not fully abolish tanshinones’ anti-cancer function, there should be multiple targets in multiple steps involved to the effects of tanshinones inhibition of tumor progression^50^. Recently, Li et al.reported that TSA docked into the ATP-binding pocket of Aurora B, and was a potential ATP competitive kinase inhibitor^51^. However, that study lacked evidence of direct binding between TSA and Aurora B kinase. It will be interesting to clarify the engagement between TSA and Aurora B and the potential connection to FHIT in the future work.

We also found that STS and TSA are very different on cytotoxicity for HCT116 cells, in that TSA significantly inhibited cell growth at 2 µM, while STS did not show any cytotoxicity even at 100 µM (Fig. S3A), although they are structurally similar and function interchangeable. This was also confirmed by the cloning formation assay, TSA at 1 µM clearly abolished the colony formation of HCT116 cells while no effect was detected with STS up to 10 µM (Fig. S3B). This difference is consistent to previous studies, in that Song et al.reported that TSA inhibited the proliferation, migration, and invasion of colorectal cancer^52^, osteosarcoma cells^53^, non-small cell lung cancer cells^54^, while few report indicated the anti-cancer activity of STS^38^, and Liu et al. claimed that STS was not effective on the growth and metastasis of Lewis carcinoma^55^. One explanation for this difference could be that the higher lipophilicity of TSA facilitates its uptake by cancer cells^56^.

Finally, an important finding in our study is that STS inhibits FHIT Ap3A hydrolase activity. Diadenosine polyphosphates (ApnAs) have been proposed to have several intracellular functions as signaling stress responses^18^. Indeed, Ap3A plays an essential role in platelet regulation, which provides a source of long-lasting ADP at the site of vascular injury^57^. What’s more, the intracellular concentration of Ap4A increases upon oxidative stressors, and aminoglycoside antibiotics induce the production of Ap4A in bacteria, which in turn enhances their bactericidal activity^58^. In addition, Ap4A attenuates STING-dependent signaling and inflammatory^59^. Given that tanshinone compounds have similar activities, such as anticoagulant, anti-oxidant, and anti-inflammatory^24^, tanshinone compounds directly bind on FHIT ‘active site’ and inhibit its hydrolase activity may shed light on the molecular mechanism of the function for those compounds. The role of FHIT and its hydrolase activity in cardiovascular, ROS, and STING signaling is also worth to be anticipated in future studies.

## Materials and methods

### Chemicals

Sodium tanshinone IIA sulfonate (STS), Tanshinone IIA (TSA), tanshinone I (TanI), and Cryptotanshinone (CST) are purchased from APExBIO.

### Cell culture

The HCT116 CRC cell line is purchased from the American Type Culture Collection (Manassas,VA). HCT116 cells were cultured in RPMI 1640 medium (Thermo Fisher Scientific). The medium was supplemented with 10% fetal bovine serum (FBS) (Gibco, Gaithersburg, MD) and 1% penicillin − streptomycin (Hyclone, Logan, UT), and the cell lines were maintained at 37 °C in a humidified atmosphere consisting of 5% CO2 and 95% air.

### Protein purification

The gene encoding human FHIT (P49789) was synthesized (Genscript, China) and cloned into pGEX-6P-1 such that the expressed protein would have an N-terminal glutathione S-transferase (GST)-tagged protein. The construct was transformed into expression host *E.coli* BL21 (DE3), and cells were grown in LB medium at 37 °C until the OD600 reached 0.6. The cells were then induced by adding 0.1 mM isopropyl-β-D-l-thiogalactopyranoside (IPTG) and grown at 18 °C overnight. Cells were harvested by centrifugation. The cell pellet was resuspended in a buffer containing 20 mM Tris (pH 7.5), 500 mM NaCl, and 2 mM DTT and lysed by sonication. After centrifugation, the clarified cell lysate was incubated with glutathione-sepharose 4B beads, and GST-tagged FHIT was eluted with the buffer consisting of 50 mM reduced glutathione. GST tag was cleaved by prescission protease, and the released FHIT was further purified to homogeneity by successive chromatographic steps involving glutathione-sepharose 4B beads, and gel- filtration columns. Pure protein fractions in buffer containing 20 mm Tris (pH 7.5), 150 mM NaCl, and 2 mM DTT were collected and used for the indicated assays. The single-site mutants H96N of FHIT was also constructed in the same vector using two primers, with required mutation using PCR. The mutant was purified with the same protocol as the wild-type FHIT. The gene encoding human HINT1 (P49773), GALT (P07902), DCPS (Q96C86) were synthesized (Genscript, China) and cloned into pET-28a such that the expressed proteins would have an N-terminal 6 × His-tag. Those plasmids were expressed in *E. coli* BL21(DE3) strain, purified as recommended before, by Co-NTA beads, then size exclusion (Superdex200 increase 10/300GL), and Anion exchange (ENrich Q) columns case by case. Finally, all the proteins were concentrated in 20 mM Tris (pH 7.5), 150 mM NaCl, 2 mM DTT, and stored at − 80 °C.

### Microscale thermophoresis (MST)

Ten million 293 cells overexpressing GFP-tagged FHIT or free GFP were lysed in 0.5 mL M-PER mammalian protein extraction reagent (Thermo Fisher Scientific). Cell lysates were diluted in PBS buffer to a final concentration at which the fluorescent signals of the GFP proteins were suitable for the detection of the Monolith NT.115 instrument (NanoTemper Technologies). A single point screening assay was developed against GFP-FHIT protein to find potential binding molecules from compound libraries (L1021,APExBIO). For binding affinity detection, a 10µL protein sample was mixed with 10µL ligand at various concentrations as indicated. And the purified proteins were labeled by Red- NHS kit in accordance with the manufacturer’s protocol (MO-L011, NanoTemper Technologies). Then the mixture solutions were loaded into NT.115 standard coated capillaries or premium coated capillaries (NanoTemper Technologies), and the MST measurements were performed at 25 °C. The fluorescence signal during the thermophoresis was monitored and the change in fluorescence was analyzed by software. The Kd was calculated by fitting a standard binding curve to the series diluted ligands. And data were analyzed by GraphPad Prism 7.0.

### FHIT hydrolase activity detection assay

5 nM FHIT protein was mixed with series diluted compounds as indicated in assay buffer (20 mM Tris–HCl, 2 mM DTT, 5 mM MgCl2, pH7.5) in a final DMSO concentration of 1% in a total volume of 20 μL. The mixtures were incubated for 30 min at 25 °C, then 500 nM Ap3A was added. After incubation at 25 °C for 5 min, the production of ADP in the reaction system was detected by ADP-Glo reagent (V6930, Promega) according to the manufacturer’s protocol in 384-well. Luminescence was read by spark (TECAN), an integration time of 1 s per well. And the data were analyzed by GraphPad Prism 7.0.

### Fluorescent thin layer enzyme assay

Fluorescent substrates ApppBODIPY at 5 µM were incubated with purified Fhit protein 10 nM in reactions at 25 °C containing 20 mM Tris–HCl, pH7.5, 150 mM NaCl, and 5 mM MgCl2. Competitive compounds, when indicated at series concentrations, were mixed with a fluorescent substrate. Reaction samples (0.6µL) were spotted on silica TLC plates (MERCK) at 60–120 s intervals. Plates were air-dried and developed in 2-propanol:NH4OH:1,4-dioxane: H2O (50:35:8:7) as reported before^37^. Developed plates were imaged by fluorescein blot (ChemiDoc, Bio-Rad).

### Molecular docking

To find STS binding site against FHIT, the X-ray crystal structure of FHIT with AMP (PDB ID: 3FIT) was downloaded from Protein Data Bank (PDB). The protein structure was prepared using AutoDockTools-1.5.6, including deleting AMP and water molecules, and adding hydrogens, before the corresponding protein grid files were generated for docking. Then the structure file of STS was pretreated in the Ligand module of AutoDockTools-1.5.6, and docking was performed with AutoDock Vina software as mentioned before^60^. The docking pose for the FHIT–STS complex was then submitted to binding mode analysis and figure generation using PyMOL1.8.

### Fluorescence polarization assay

The binding of FHIT (H96N) to fluorescein-labeled substrates ApppBODIPY was analyzed by fluorescence polarization assay in 384-well format for dose–response curve studies using spark instrument (TECAN) according to the protocol reported before^61^. And 5 µM FHIT (H96N) protein was incubated with 1 µM fluorescein-labeled substrates in buffer A (25 mM Tris–HCl, pH 7.5, 5 mM MgCl2 and 2 mM DTT) with series diluted inhibitors as indicated. Each sample was allowed to equilibrate in the solution for 30 min. After 30 min, the steady-state fluorescence anisotropy (mP) was measured. A second reading was taken after 10 min, to ensure that the mixture was well-equilibrated and stable. Less than 5% change was observed between 30 and 40 min measurements. The IC50 value was determined by fitting the binding curves using GraphPad Prism 7.0.

### CCK-8 assay for cell inhibitory rate

The CCK-8 (Cell-Counting Kit-8) assay was used to detect the cells’inhibitory rate following the manufacturer’s protocol (APExBIO). Cells were seeded into 96-well plates at the density of 4 × 10^3^(cells/well) overnight at 37 °C. After that, the culture medium was removed and the cells were treated with different concentrations of TSA (0, 1, 2, 5, 10 µM) or STS (0, 10, 25, 50, 100 µM) for 24 h, 48 h, 72 h respectively. Then 10 μL CCK-8 reagent was added into each well and incubated for another 2 h, OD450 was measured by a microplate reader (spark, TECAN).

### Western Blot analysis

Cells were lysed using RIPA (Cell Signaling Technology) containing protease inhibitor (Thermo Fisher Scientific) at 4 °C. Proteins were quantified by BCA protein quantification kit (Thermo Fisher Scientific). And proteins (10–30 μg per lane) were separated by SDS-PAGE and then transferred onto polyvinylidene fluoride membranes (Millipore). Following this, the membranes were blocked with 5% non-fat milk for an hour. Later on, the membranes were incubated with primary antibodies anti-FHIT (#ab170888, Abcam), anti-GAPDH (#5174, CST) overnight at 4 °C. After that, the membranes were incubated with HRP-goat anti-mouse secondary antibodies (1:5000, CST) for 1 h, Enhanced chemiluminescence detection reagent (NCM biotech) was used to visualize the signal strength of the bands.

### CRISPR/Cas9 mediated FHIT knockout assay

*FHIT* gene was deleted in HCT116 cells by CRISPR/Cas9 genome editing. A plasmid pL- CRISPR.EFS.GFP (#57,818, Addgene) encoding both the Cas9 protein and the sgRNA was used. The Cas9 sequence is coupled to a P2A site and EGFP. Expression of the Cas9 protein results in simultaneous expression of EGFP, allowing for the selection of positively transfected cells. Two sgRNA toward exon5 and exon6 of the FHIT gene, respectively, were designed by using the optimized CRISPR design online tool (http://crispr.cos.uni-heidelberg.de). The primers sequences of the sgRNA used were shown as below: sgRNA1 for exon5 forward, 5’CACCGTGTCCTTCGCTCTTGTGAAT3’, reverse, 5’AAACATTCACAAGAGCGAA GGACAC3’; sgRNA2 for exon6 forward, 5’CACCGACCCAGAGAGTCGGGACAG3’, reverse, 5’AAACCTGTCCCGACTCTCTGGGTC3’. Plasmids containing the right insertion were then sequenced by Sangon Biotech Company (Shanghai, China). The two CRISPR/Cas9 plasmids were co-transfected into HCT116 cells as 1:1 using Lipofectamine3000 (Invitrogen). After 48 h, EGFP positive single cell was sorted into 96-well plates and the plates were left in the incubator for 7–14 days or until small colonies could be seen by the naked eye. Genomic DNA of each well was extracted by QuickExtract DNA Extraction Solution (Epicenter, Madison, WI) to do PCR identification. And the PCR products were then Sanger-sequenced to identify clones that would result in frameshifts and truncated protein products. Sequence alignment and genomic PCR primers were shown as below: forward, 5′GAATCCATCTCCCTCCCA3′, revers, 5′CATCACAAAGGCACCACTC3′.

### Apoptosis detection by flow cytometry analyses

HCT116 cells treated with indicated compounds were digested with 0.25% trypsin (Gibco). The cells were harvested by centrifugation at 1000 rpm for 5 min. The trypsinization time should not be too long to prevent false positives. The cells were washed twice with pre-cooled PBS. The cells were resuspended with 1 × annexin-binding buffer and the cell density was adjusted to ~ 1 × 10^6^ cells/ml. Then 5µL Alexa Fluor 488 annexin V and 1 µL 100 µg/mL PI working solution (V13241, Invitrogen) was added to each 100 µL cell suspension. The cells were incubated at room temperature in the dark for 15 min. After the incubation, 400 µL 1 × annexin-binding buffer was added to the cells, mixed gently, and kept the samples on ice. Analyze the stained cells by flow cytometry as soon as possible. The V-FITC positive and PI negative cells were recorded as apoptotic cells.

### Statistical analysis

The results are expressed as the mean ± SEM. The data analysis was performed using GraphPad Prism version 7.

## Supplementary Information

Supplementary Information.

## Data Availability

All data pertinent to this work are contained within this manuscript or available upon request. For request, please contact: Innovation Center of Chemical Biology, Institute of Interdisciplinary Integrative Medicine Research, Shanghai University of Traditional Chinese Medicine, Shanghai, China, xisongke@shutcm.edu.cn.
