# Supplementary material for: Tanshinones induce tumor cell apoptosis via directly targeting FHIT
Source: Sci Rep. 2021 Jun 9;11:12217. doi: 10.1038/s41598-021-91708-z (PMC8190080; doi:10.1038/s41598-021-91708-z)
Supplement: Supplementary file 1 — Supplementary Information. [file 41598_2021_91708_MOESM1_ESM.pdf]

Supplemental Information

Tanshinones induce the tumor cell apoptosis via directly targeting FHIT

Xianglian Zhou[1], Yuting Pan[1], Yue Wang[1], Bojun Wang[1], Yu Yan[2], Yi Qu[1*], Xisong Ke[1*]

Materials and methods

ENPP1 hydrolase activity detection assay

ENPP1 protein (4ng/μl) was mixed with series diluted compounds as indicated in assay buffer (50mM Tris-HCl, 2mM CaCl2, 2mM MgCl2, pH9.0) in a final DMSO concentration of 1% in a total volume of 20μL. The mixtures were incubated for 30min at 25°C, then 4μM Ap3A was added. After incubation at 25°C for 10min, ENPP1 should be inactivated at 85°C for 5min. Then the production of ADP in the reaction system was detected by ADP-Glo reagent (V6930, Promega) according to the manufacturer's protocol in 384-well. Luminescence was read by spark (TECAN), an integration time of 1 second per well. And the data were analyzed by GraphPad Prism 7.0.

Fluorescent thin layer enzyme assay for ENPP1

Small molecules, when indicated at series concentrations, were mixed with 2.5μM the fluorescent substrate ApppBODIPY. Then, 10nM purified ENPP1 protein was added to the reaction system at 25°C containing 50mM Tris-HCl pH9.0, 2mM $CaCl_2$, 2mM $MgCl_2$. Reaction samples (0.6μL) were spotted on silica TLC plates (Merck) at 60–120s intervals. Plates were air-dried and developed in 2-propanol:NH_4OH:1,4-dioxane: H_2O (50:35:8:7) as reported before[1]. Developed plates were imaged by fluorescein blot (ChemiDoc, Bio-rad).

Colony Formation Assay

Colony formation assay was performed as described before[2]. Briefly, ～200/well single cells were planted into 6-well plate. Cells were treated with small molecules at indicated concentration for 2-weeks, the colonies were fixed and stained by PBS containing 4% formaldehyde and 0.005% crystal violet.

References

1. Draganescu, A., *Fhit-nucleotide Specificity Probed with Novel Fluorescent and Fluorogenic Substrates.* Biochem J, 2000.
2. Crowley, L.C., M.E. Christensen, and N.J. Waterhouse, *Measuring Survival of Adherent Cells with the Colony-Forming Assay.* Cold Spring Harb Protoc, 2016. **2016**(8).

Supplemental Figures

Figure S1

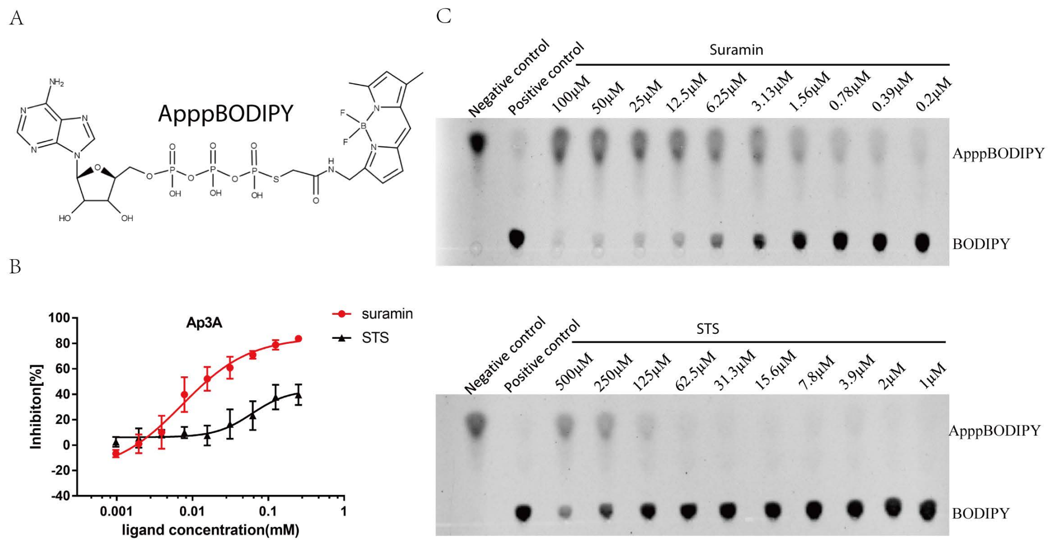

Supplementary Figure S1. Related to Figure 4A. STS inhibits ENPP1 Ap3A hydrolase activity at very high concentration. (A) Chemical structure of ApppBODIPY used for fluorescent thin layer enzyme assay and fluorescence polarization assay. (B) ENPP1 Ap3A hydrolase activity assay. black triangle indicates the inhibition of STS with IC_{50}=57.87±15.13μM, red circle indicates Suramin with IC_{50}=8.3±3.54μM. (C) Fluorescent thin layer enzyme assays of ENPP1 inhibited by Suramin (IC_{50}=6.25μM) and STS (IC_{50}=125μM).

Figure S2

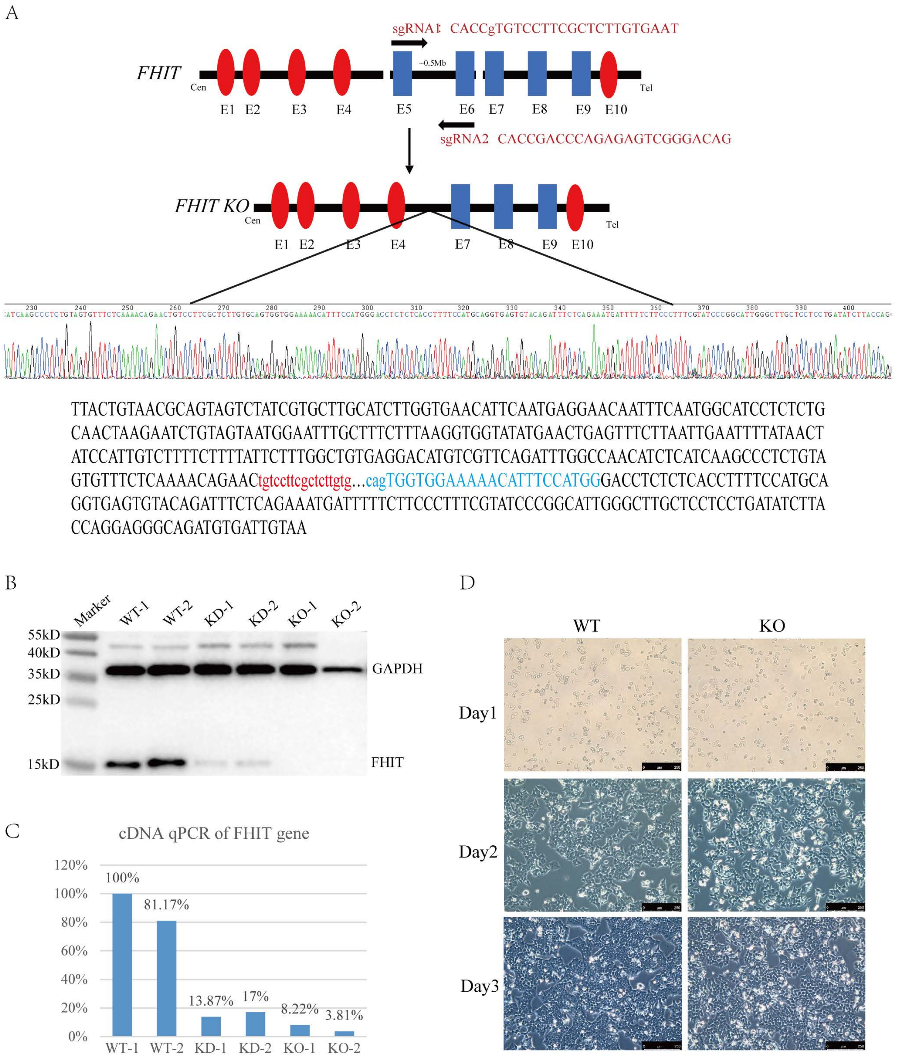

Supplementary Figure S2. Related to Figure 6. CRISPR/Cas9knockout of FHIT in HCT116 cells. (A) Schematic diagram of by CRISPR/Cas9 knockout of FHIT and sequencing confirmation. (B) Western blot analysis of FHIT abundance in FHIT KO cells. KO-1 cells were used for following work. (C) qPCR analysis of *FHIT* gene in FHIT KO cells. (D) The epithelial morphology and growth rate comparation of HCT116 wild-type (WT) cells and FHIT KO cells.

Figure S3

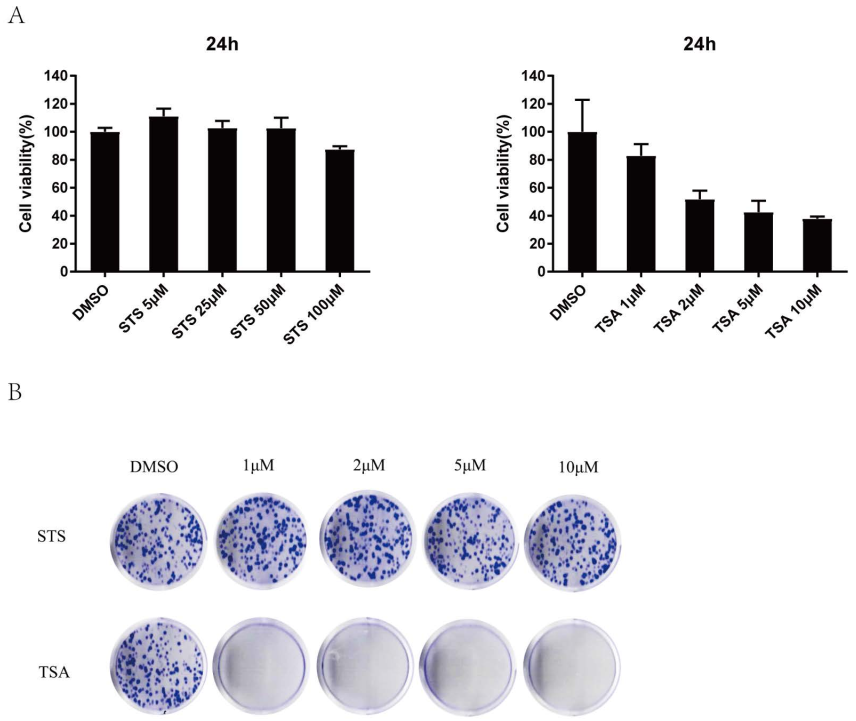

Supplementary Figure S3. Cell growth was significantly inhibited by TSA but not by STS. (A) CCK8 assays of HCT116 cells treated with STS or TSA at indicated concentration for 24 hours. (B) The effect of STS and TSA on the colony-forming of HCT116 cells, respectively. Cells were treated with DMSO or the indicated concentration of STS or TSA for 14 days and stained with crystal violet.
